# Supplementary material for: Emotional attachment and resistance to change in the use of technology: A study among Chinese university music teachers
Source: PLoS One. 2025 Jul 2;20(7):e0327205. doi: 10.1371/journal.pone.0327205 (PMC12221048; doi:10.1371/journal.pone.0327205)
Supplement: Attachment — Submitted filename: Reviewer-Comments.pdf [file pone.0327205.s003.pdf]

Dear Editor

I'd like to thank you for inviting me to peer-review the manuscript [PONE-D-24-35928R1] & to thank the author(s) for their work and effort.

As the abstract suggests, the research addresses resistance to change as and emotional attachment as explanatory variables for using technology in teaching music in Chinese universities.

In my opinion, the effort exerted in this research needs to be complemented by deeper analysis and restructuring.

To help improve the manuscript, I'd like to share some comments & recommendations:

1) Starting p.9, it is noted that all hypotheses are non-directional.
My recommendation is to opt for directional hypotheses as, in scientific research, they are highly preferred to non-directional ones. Directional hypotheses provide precise theoretical expectations derived from literature, which are important for developing rigorous research.

2) In P.9-10, two points (2.3. emotional attachment) and (2.4. resistance to change), they do not include how both constructs are defined.
Still, in P. 20-21, there are definitions of both constructs.
It is standard and highly recommended that nominal definitions of key concepts are mentioned either in the theoretical part or in the description of variables where concepts are operationalized. In either case, definitions should be added before data analysis and discussion.

3) In Pp 13-14, it is stated that "Initially, some music teachers were contacted by the authors and subsequently, more teachers were invited to participate in this project". This sentence left me convinced that convenience sampling was combined with snowballing sampling.
If this is the case, I recommend that you state it clearly.

4) When was the survey fielded? The answer to this question is important to know for two reasons:
First, stating data collection time frame is conventionally part of the research design.
Second, if the survey was fielded after COVID-19, this entails that using technology is already established as a teaching method for all disciplines.
In p. 21, it is stated that "Music teachers may perceive that using technology in teaching has become the norm", which raises questions about the existence of

"resistance to change" and its usefulness as an explanatory variable.
My recommendation is to clarify for the reader why- if using technology is the norm- speaking of resistance still matters in explaining technology adoption in music teaching.

5) Relatedly, the article's theoretical outlook and research design considered that "resistance to change" might be correlated to "the perceived ease/difficulty of use".
However, in p.21 it explained resistance to change in reference to the difficulty of changing habits- which is not exclusive to music teachers but applies to individuals at large.
This leads me to asking: have your research control for personal traits and predispositions related to openness and flexibility to change?

6) In the article, participants' demographic summaries are provided in P.13 under the sub-title (participants), and under the title (results) in p. 16, there is a sub-title for (descriptive analysis).
I recommend that the authors re-structure the article in a way that complies with the standards of writing a quantitative paper where- after the theoretical part ending with the hypotheses- there is a title for
Research Design, under which the following parts should be mentioned:
- data collection tools and administration, IRB approval, ethical considerations...etc.
- sampling procedure
- How the dependent variable is operationalized & measured in the survey
- How the independent variables are operationalized & measured in the survey.
- The control variables (are essentially the demographic variables mentioned)
- Descriptives/Summary Statistics (which are essentially what is mentioned in p.16 under the title descriptive analysis)
- Empirical model estimation

The article has all these required parts but scattered in different places. Thus, the article will benefit from thorough restructuring.

7) For in Pp.14-15: in the 7-point Likert scale, the fourth statement is "I will use technology in my future music teaching" is used to elicit "behavioral intention"- which is thought to be the dependent variable, as depicted in figure 2-
This is a future-oriented sentence, yes, but should have been analyzed in relation to who is currently (at the present) using technology and who is not?
In retrospect to the participants, asking about (years of teaching with technology) have values between 0-40, with average of 5.77 years and St.Dev= 6.

(P.13, table 1), which means that the coefficient of variance (st.dev/the mean) is almost =1, indicating a high variability between respondents in this regard.
So, the phrasing of the questionnaire is vague because it does not differentiate between those who have adopted technology and those who have not.
In the analysis, there should be two clusters at least: those who have not used technology at all, those who have used it.
Then, sixth sentence on the Likert scale (I do not expect to change my teaching method)- which has a future-oriented connotation- will make sense for those eliciting resistance to change for those who have NOT used technology at all, and will be meaningless for those who have already done (because they did not resist).
I have two recommendations in this regard:
a) the author(s) should share a copy of the survey's English version, as part of the supporting material to help better evaluate what can be done with it.
b) the authors should re-do the analysis to compare between both groups (those who have used tech.) and (those who did not)—with special focus on those who did not.
If the number of participants under the category of (those who did not use technology) is not large enough for statistical analysis as per the central limited theorem, there might be a need to resample (those who did not use technology at all) OR preferably (those who reverted back to old ways of teaching music after COVID-19 ended)- because, as mentioned before, it is not imaginable that anyone did not use technology since COVID-19 outbreak.

8) I would recommend that you add a sub-section for robustness checks where you test your results after controlling for gender over-representation in the sample (almost 63 % female, while 37% male), especially for the emotional attachment explanation; and for the type of university (top-ranked, or not) because this factors in the level of institutional infrastructure and resources available for students and teachers in regard with the use of technology in teaching.

9) Typo: P. 17: "model ft" should be "model fit".

10) Pp18-19, in regard with the discussion of the regression coefficients, I recommend that you interpret the results in terms of the size, significance and direction of the correlation.
I also would recommend that you opt for the standard way of presenting output results for the analysis.

11) In the path diagram (figure 2, page 12) and the analysis & discussion, there should be something said about the un-theorized and un-analyzed correlation

between emotional attachment and resistance to change (depicted as a curved two-headed arrow in figure 2).

In some sense, and when liking this to the nominal definitions of both terms, having emotional attachment to old ways of teaching, might make the individual anxious or stressed about the new way, which contributes to “resistance to change”. Now, with this understanding, a number of questions emerge:

is the relationship between emotional attachment and resistance to change spurious? What can be said of their correlation for the analysis?

Is it better to include both in the model? OR it is enough to re-conceptualize “resistance to change” as a composite index including

personal traits of openness, ease/difficulty of using the new tools, being emotionally attached to the old teaching approaches?

I recommend the latter as a solid analytical option that will reduce the number of variables involved by creating a composite index, leading to fewer hypotheses and a simpler model.

Once again, thank you and the authors for sharing the manuscript with me.

Sincerely
