# Supplementary material for: Emotional attachment and resistance to change in the use of technology: A study among Chinese university music teachers
Source: PLoS One. 2025 Jul 2;20(7):e0327205. doi: 10.1371/journal.pone.0327205 (PMC12221048; doi:10.1371/journal.pone.0327205)
Supplement: Attachment — Submitted filename: Response.docx [file pone.0327205.s004.docx]

**Response to Reviewers**

MS Title: Emotional attachment and resistance to change in the use of technology: A study among Chinese university music teachers

| **Comments from editors** | **Responses** |
| --- | --- |
| We note that the grant information you provided in the ‘Funding Information’ and ‘Financial Disclosure’ sections do not match. When you resubmit, please ensure that you provide the correct grant numbers for the awards you received for your study in the ‘Funding Information’ section. | We have revised the grant information, thank you for your reminding. |
| All PLOS journals now require all data underlying the findings described in their manuscript to be freely available to other researchers, either 1. In a public repository, 2. Within the manuscript itself, or 3. Uploaded as supplementary information. The data should be provided as part of the manuscript or its supporting information, or deposited to a public repository. | We have supplied the data when we submit the revised version. |
| PLOS requires an ORCID iD for the corresponding author in Editorial Manager on papers submitted after December 6th, 2016. | The ORCID (0009-0008-8564-5452) is provided in the text we submitted, thank you. |
| Please ensure that you include a title page within your main document. You should list all authors and all affiliations as per our author instructions and clearly indicate the corresponding author. | We have added a title page including authors’ information in the main document, thank you. |
| Please amend either the abstract on the online submission form (via Edit Submission) or the abstract in the manuscript so that they are identical. | We amended the abstract to make sure they are identical, thank you. |
| Please include your full ethics statement in the ‘Methods’ section of your manuscript file. In your statement, please include the full name of the IRB or ethics committee who approved or waived your study, as well as whether or not you obtained informed written or verbal consent. If consent was waived for your study, please include this information in your statement as well. | Thank you for your suggestion. We have added contents to describe the IRB and informed consents in the method section (page 12). |
| **Comments from Reviewer 1** | **Responses** |
| Introduction: It would be advisable to include at the end of the introduction the general objective, the specific objectives and the study hypotheses with their corresponding citations from the studies on which the authors have based these hypotheses. | Thank you, we have added the objectives of the study at the end of the introduction. Hypotheses were rationalized in the literature review section, and this is a quite standardized way of presenting hypotheses, so please allow us to remain this style.   - Page 5:...Therefore, the current study aims to unpack music teachers’ perceptions of adopting technology in music teaching, and specifically, the extend to which the proposed research model based on the technology acceptance theory explained music teachers’ technology using intentions.... |
| Instruments: An item example should be collected for the variables used. In addition, it should be explained whether the validity and reliability of the instrument was assessed as a single instrument or whether it was assessed by factors, taking into account that they are items of different instruments. | Thank you, we have added item example and descriptions of the validity and reliability of the factors we have used (Page 14). |
| Data analysis: The explanation of data analysis should be expanded and criteria added to specify whether the results obtained are adequate according to the test performed. Procedure: A procedural sub-section should be added, setting out how the questionnaire was administered, when, how long it lasted and ethical considerations of the study. | We elaborated on the data analysis, especially the criteria (please refer to revision in the Page 15).  In addition, a sub-section named “procedure” was presented following by a section of “participants”, as you suggested (please refer to Page 13). |
| Results: If the instrument is set up as a single instrument, an exploratory factor analysis should be carried out beforehand. If different instruments are validated, it should be clarified that different confirmatory factor analyses were performed and explain all this information in the section of instruments (by test) and in the section of data analysis. - Table 3 needs to be explained in more detail. | Thank you, the instrument was set up as a single instrument, we added an EFA result in the text (page 16) .  We also explained more about the table 3 in the text (page 18). |
| Discussion: - The discusión should be extended, hypothesis to hypothesis and whether it is rejected or accepted and its justification. | We have extended the discussion section (Page 19-21). Thank you for your suggestion. |
| References: - Review the appointments. - Review references to the journal’s standards. | Thank you, references were reviewed. |
| **Comments from Reviewer 2** | **Responses** |
| Sampling and Participant Characteristics: The study states that convenience sampling was used to recruit participants from 52 Chinese universities, but more details on the recruitment process would be beneficial. o Were there any inclusion or exclusion criteria? o Certain factors that could influence the results, such as teachers’ age, years of teaching experience, and years of teaching with technology, do not seem to have been considered. While these aspects are mentioned as descriptive variables, their potential impact on technology acceptance is not discussed. Although this may fall outside the primary goal of the study, acknowledging these as possible influencing factors would strengthen the analysis. o Additionally, was regional diversity taken into account (e.g., differences between South and North China)? This factor could also play a role in technology acceptance. | Thank you, we have used a sub-section titled “procedure” to report the details,including the recruitment criteria. Please refer the Page 13 for the details.  We do agree with your opinion that some demographic factors and location would also influence thinking and behavior, but considering the main aim of the study and the strict word limit, we decided to focus on the roles of emotional attachment and resistance to change in influencing music teachers’ technology adoption.  We do provide suggestions for further study to consider the roles of these factors, please refer to our revision in the text (Page 22). |
| Definition and Scope of Technology in Music Teaching: The study discusses various ways in which technology can enhance music teaching—ranging from specialized software to social networks, and remote teaching (tele-teaching). However, it is not clear which specific types of technology the study refers to. o Different technological tools may serve different teaching purposes, and their levels of acceptance may vary accordingly. o In particular, for the administered questionnaire, it would be helpful to specify which technologies were considered and what the participants were asked to evaluate. Providing clarification on these points would further strengthen the study’s contribution and make its findings more interpretable. | Thank you for your suggestion. Online teaching is the context we are looking at.  According to teachers’ responses, the tools they use for online teaching mainly include DingTalk, Tencent Meeting, QQ, and WeChat, etc. These are well-acknowledged technology tools in China. We have added this information in the Method section (page 13).  We appreciate your suggestion of thinking of the difference in tools, and we put them in the suggestions for further study (Page 22). |
