# Supplementary material for: Emotional attachment and resistance to change in the use of technology: A study among Chinese university music teachers
Source: PLoS One. 2025 Jul 2;20(7):e0327205. doi: 10.1371/journal.pone.0327205 (PMC12221048; doi:10.1371/journal.pone.0327205)
Supplement: Attachment — Submitted filename: Response letter.docx [file pone.0327205.s005.docx]

**Response to reviewers**

| Reviewer #2: | Response |
| --- | --- |
| I thanks the authors to have addressed my comments and provided the requested clarifications.I believe that the manuscript is now ready for publication in its current version.  Good luck! | Thank you for your support. |
| Reviewer #3 | Response |
| First, a link to the dataset is not currently present, and a dataset was not attached with the reviewer copy. There is no indication that these data are publicly available separately from the manuscript. Either way, the authors need to supply a link to replication data and code. If a dataset was supplied and supposed be attached, disregard. | We have added Data availability statement in the text (page 25), thank you. |
| Second, I would suggest the authors put their RQs in bullet point form, and to rephrase RQ1 so it's clearly referencing the Technology Acceptance Model. | Thank you for your suggestions, we have accordingly change the presenting style into bulletin form (page 5) |
| Third, the hypotheses are technically correct but uninformative. If the authors expect one variable to have a positive/negative relationship with another, there is nothing wrong with stating that. It doesn't affect the analyses, but it's immensely helpful to the reader. I'm sure the original theory proposed directional hypotheses. I would also suggest using full variable names instead of abbreviations to add clarity. | Thank you. We clarified the positive or negative relationships in the hypotheses for those developed from theory.  We also use the full names for the variables which are easier for the readers to understand. Please refer to our revision in the text. |
| Fourth, if Figure 2 is the model being tested, the authors do not need Figure 1. | Thank you, we have removed figure 1 from the text. |
| Fifth, when discussing measurement, it would be easier if the authors presented one variable at a time, paragraph by paragraph (similar to how social psychologists would present it). That way, all the information about a variable (number of items, example of an item, reliability) is in one place. | We took your suggestion and present variables one by one, using different paragraphs (page 14) |
| Sixth, it is more common to use Cronbach's alpha or fit statistics from a Confirmatory Factor Analysis to assess how well a variable fits to the data. I would suggest the authors use either one of those when discussing reliability/data fit instead of Composite Reliability or Average Variance Explained. | When doing structural equation modeling, it is a norm to use CR and AVE to report reliability and validity. But for readers’ sake, we also reported values of cronbach alpha (page 17). |
| Seventh, when discussing results, it is helpful to actually state what the results were beyond whether they were significant. What was their direction? Does the finding support the hypothesis? Yes, it can be seen from Table 3, but it is worth stating to the reader verbally as well. | We have revised the discussion section based on your suggestions. Please refer to our revision in the text (page 19-21). |
| Eighth, the coefficient testing H8 is missing a digit. | Thank you, we have added the digit in the text (page 19). |
| Reviewer #4 | Responses |
| Starting p.9, it is noted that all hypotheses are non-directional. My recommendation is to opt for directional hypotheses as, in scientific  research, they are highly preferred to non-directional ones. Directional hypotheses provide precise theoretical expectations derived from literature, which are important for developing rigorous research. | Thank you. Following your suggestions, We have indicated positive or negative relationships in the hypotheses except H8, since we could not ensure its direction, but the result suggested its positive direction, and we have provided discussion on it (page 22). |
| In P.9-10, two points (2.3. emotional attachment) and (2.4. resistance to change),  they do not include how both constructs are defined. Still, in P. 20-21, there are definitions of both constructs. It is standard and highly recommended that nominal definitions of key concepts are mentioned either in the theoretical part or in the description of variables where concepts are operationalized. In either case, definitions should be added before data analysis and discussion. | Thank you for your suggestions. We have added their operational definitions in both the literature review section (page 10 & 11) and survey section (page 15), in the text. |
| In Pp 13-14, it is stated that “Initially, some music teachers were contacted by  the authors and subsequently, more teachers were invited to participate in this  project”. This sentence left me convinced that convenience sampling was  combined with snowballing sampling.  If this is the case, I recommend that you state it clearly | Thank you for your informative suggestion, we revised this part in the text (page 14). |
| When was the survey fielded? The answer to this question is important to know for two reasons:  First, stating data collection time frame is conventionally part of the research design.  Second, if the survey was fielded after COVID-19, this entails that using  technology is already established as a teaching method for all disciplines.  In p. 21, it is stated that “Music teachers may perceive that using technology in  teaching has become the norm”, which raises questions about the existence of  “resistance to change” and its usefulness as an explanatory variable.  My recommendation is to clarify for the reader why- if using technology is the  norm- speaking of resistance still matters in explaining technology adoption in  music teaching. | We really appreciate your suggestions. We added descriptions to indicate the time of the data collection (page 14 ). It is worth noticing that technology acceptance study entails willingness to use technology, and its value is to measure the degree to which some factors influence people’s thinking, no matter if they have used technology or not. This can be witnessed in many technology acceptance studies in literature. Therefore, we believe it is still proper and valuable to understand teachers’ technology using intentions, and know more about the degree to which some important psychological factors influence their intentions.  In the text, we added rationalization for resistance to change, and clarified that it measures their willingness to innovate existing pedagogy (page 11). |
| in p.21 it explained resistance to change in reference to the difficulty of changing habits- which is not exclusive to music teachers but applies to individuals at large.  This leads me to asking: have your research control for personal traits and predispositions related to openness and flexibility to change? | Thank you, we did not control personal traits in this study, but yes, we may measure personal traits in future studies to know about if personal traits influence resistance to change. We put it in the limitations and suggestions for future study section (page 24). |
| I recommend that the authors re-structure the article in a way that complies with  the standards of writing a quantitative paper where- after the theoretical part  ending with the hypotheses- there is a title for  Research Design, under which the following parts should be mentioned:  - data collection tools and administration, IRB approval, ethical  considerations…etc.  - sampling procedure  - How the dependent variable is operationalized & measured in the survey  - How the independent variables are operationalized & measured in the survey.  - The control variables (are essentially the demographic variables mentioned)  - Descriptives/Summary Statistics (which are essentially what is mentioned in p.16 under the title descriptive analysis)  - Empirical model estimation | Thank you, and we followed your suggestions and renamed method to research design. Please allow us to remain “participants”,”Procedure”... as they are also standardized in educational and psychological studies. We ensure that details of ethical approval has been introduced in the research design section (page 12).  We have added clarifications in this section (page 14-15). Please allow us to remain descriptive results in the result section as we believe they belong to it, and many studies do so. |
| For in Pp.14-15: in the 7-point Likert scale, the fourth statement is “I will use technology in my future music teaching” is used to elicit “behavioral intention”- which is thought to be the dependent variable, as depicted in figure 2-  This is a future-oriented sentence, yes, but should have been analyzed in relation to who is currently (at the present) using technology and who is not?  In retrospect to the participants, asking about (years of teaching with technology) have values between 0-40, with average of 5.77 years and St.Dev= 6.(P.13, table 1), which means that the coefficient of variance (st.dev/the mean) is  almost =1, indicating a high variability between respondents in this regard. So, the phrasing of the questionnaire is vague because it does not differentiate between those who have adopted technology and those who have not.  In the analysis, there should be two clusters at least: those who have not used  technology at all, those who have used it.  Then, sixth sentence on the Likert scale (I do not expect to change my teaching method)- which has a future-oriented connotation- will make sense for those eliciting resistance to change for those who have NOT used technology at all, and will be meaningless for those who have already done (because they did not resist).  I have two recommendations in this regard:  a) the author(s) should share a copy of the survey’s English version, as part of the  supporting material to help better evaluate what can be done with it.  b) the authors should re-do the analysis to compare between both groups (those who have used tech.) and (those who did not)—with special focus on those who  did not. If the number of participants under the category of (those who did not use  technology) is not large enough for statistical analysis as per the central limited theorem, there might be a need to resample (those who did not use technology at all) OR preferably (those who reverted back to old ways of teaching music after COVID-19 ended)- because, as mentioned before, it is not imaginable that anyone did not use technology since COVID-19 outbreak. | Technology acceptance examines (1) if people use or not use technology and (2) the degree to which they are willing to use, by using behavioral intention as the endogenous variable, because it directly leads to actual usage (Davis, 1989).  We also understand there is a big difference in terms of using experience, but since the focus of the study using SEM technical is to understand the relationships, instead of gaining deep understanding of differences by cases, we did not group participants in the data analysis. Future studies could do so to see if using experience moderated some relationships. We put it in the suggestions for future study section (page 24).  Resistance to change measures their willingness to change existing ways of doing. As innovative technology always emerges, it is appropriate to be included in this study. |
| I would recommend that you add a sub-section for robustness checks where you test your results after controlling for gender over-representation in the sample(almost 63 % female, while 37% male), especially for the emotional attachment explanation; and for the type of university (top-ranked, or not) because this factors in the level of institutional infrastructure and resources available for  students and teachers in regard with the use of technology in teaching. | Thank you, we do agree that gender, institutional infrastructure and resources may influence teachers’ technology using intentions. We prefer to analyze them in the future studies. |
| Typo: P. 17: “model ft” should be “model fit”. | Sorry for this, we have revised it. |
| Pp18-19, in regard with the discussion of the regression coefficients, I recommend that you interpret the results in terms of the size, significance and direction of the correlation.  I also would recommend that you opt for the standard way of presenting output  results for the analysis. | Thank you, we have revised the discussion section as suggested. As many studies using SEM as a technique, we presented results align with existing studies. |
| In the path diagram (figure 2, page 12) and the analysis & discussion, there should be something said about the un-theorized and un-analyzed correlation between emotional attachment and resistance to change (depicted as a curved two-headed arrow in figure 2).  In some sense, and when liking this to the nominal definitions of both terms, having emotional attachment to old ways of teaching, might make the individual anxious or stressed about the new way, which contributes to “resistance to change”. Now, with this understanding, a number of questions emerge:  is the relationship between emotional attachment and resistance to change spurious? What can be said of their correlation for the analysis? Is it better to include both in the model? OR it is enough to re-conceptualize  “resistance to change” as a composite index including personal traits of openness, ease/difficulty of using the new tools, being  emotionally attached to the old teaching approaches? I recommend the latter as a solid analytical option that will reduce the number of variables involved by creating a composite index, leading to fewer hypotheses and a simpler model. | SEM requires the exogenous variables should be correlated when doing analysis. We did not build up a directional relationship between emotional attachment and resistance to change. But in the data analysis, we have to draw correlations between the two variables since SEM technique indicates variables rotate statistically.  As for the definition of resistance to change, we have provided elaborations in the text.  Thank you for your wonderful insights. We have benefited a lot. |
